# How to calculate sample size in animal and human studies

**DOI:** 10.3389/fmed.2023.1215927

**Published:** 2023-08-17

**Authors:** Xinlian Zhang, Phillipp Hartmann

**Affiliations:** ^1^Division of Biostatistics and Bioinformatics, Herbert Wertheim School of Public Health and Human Longevity Science, University of California, San Diego, La Jolla, CA, United States; ^2^Department of Pediatrics, University of California, San Diego, La Jolla, CA, United States; ^3^Division of Gastroenterology, Hepatology and Nutrition, Rady Children's Hospital San Diego, San Diego, CA, United States

**Keywords:** sample size calculation, power, effect size, animal and human study, two sample comparison, type I error, type II error

## Abstract

One of the most important statistical analyses when designing animal and human studies is the calculation of the required sample size. In this review, we define central terms in the context of sample size determination, including mean, standard deviation, statistical hypothesis testing, type I/II error, power, direction of effect, effect size, expected attrition, corrected sample size, and allocation ratio. We also provide practical examples of sample size calculations for animal and human studies based on pilot studies, larger studies similar to the proposed study—or if no previous studies are available—estimated magnitudes of the effect size per Cohen and Sawilowsky.

## Introduction

The sample size refers to the number of patients or animals included in a study, and it is one of the first and foremost questions to be answered when designing a human or animal study. It is easy to understand that a sample size smaller than necessary would result in insufficient statistical power to answer the research question and reduce the chance of reaching statistical significance. However, the choice of the sample size also does not necessarily mean the bigger the better. A large sample size will better represent the population and will hence provide more accurate results. However, the increase in accuracy will be small and clinically irrelevant after a certain point and hence not worth the effort and cost. In some studies, an excessively large sample size would expose a more than necessary number of patients/animals to potentially toxic procedures, which would be unethical. Sample size determination depends on the study design and study aim. For most cases, sample size can be determined by hypothesis testing, so that we can reject the null hypothesis with both statistical significance and practical relevance with reasonable statistical power. These procedures must consider the size of type I and type II errors as well as population variance and the effect size of the outcome of interest. There also exist cases, such as opinion surveys, in which sample size calculation usually targets an acceptably small margin of error irrespective of statistical power, type I/II error, and effect size. We focus on the former in this study.

## Definitions

In this study, we use *x*_1_, *x*_2_, …., *x*_*n*_ to denote the *n* data points for a given variable, and we mostly consider the case of a continuous variable.

### Mean and standard deviation (SD)

The mean, or the average of all values of a specific group, $\bar{x}=\overset{n}{\underset{i=1}{\sum }}x_{i}/n$, is a summary of location. The SD describes the dispersion and variability of the variable $s=\sqrt{\overset{n}{\underset{i=1}{\sum }}{\left(x_{i\;}-\bar{x}\right)}^{2}/\left(n-1\right)}$; specifically, it measures the average deviation of the data points from the mean.

### Statistical hypothesis testing

Statistical hypothesis testing is a statistical inference tool that makes use of the data collected to determine whether there is strong evidence to reject a certain hypothesis, which we term the null hypothesis. Generally, the null hypothesis is a statement of no relevant association or effect. With a null hypothesis set up, we also have an alternative hypothesis, which supports the existence of a relevant association or effect. In this review, we focus mainly on the case of comparing the means of two groups. Then, the null hypothesis is that the means of a continuous variable in two groups are the same (μ_1_ = μ_2_). The alternative hypothesis is that there is a non-zero difference between the group means of the continuous variable. Depending on the null hypothesis, a test statistic is calculated and compared to the critical value (at a given significance level, say α= 0.05) under the null hypothesis. The test statistic is a measure of how unlikely we observe the current data given the null hypothesis being true. Usually, a larger test statistic (larger in absolute value than the critical value) means that we are more unlikely to observe the current data. Thus, we tend to accept the alternative hypothesis.

### Type I error

In statistical hypothesis testing, a type I error is the probability of rejecting a true null hypothesis, i.e., this is a “false positive” conclusion. This is the significance level (α) we choose to use in statistical hypothesis testing. Common choices of α are 0.05 or 0.01. It is worth noting that a type I error is determined prior to sample size calculation.

### Type II error and power

In contrast to the type I error, the type II error, denoted as β, in statistical hypothesis testing refers to the probability of failure to reject a false null hypothesis, i.e., this is a “false negative” conclusion. The power of a statistical test (=1 – Type II error) is the probability to detect a true association, i.e., to reject a false null hypothesis. Common choices of β are either 0.2, 0.1, or 0.05.

### Direction of effect

This refers to when to reject the null hypothesis. It is rejected in a two-tailed test if the mean of one group is different (either higher or lower; μ_1_≠μ_2_) relative to the mean of another group. In a one-tailed test, the null hypothesis is rejected if the mean of one specific group is higher than that of the other (μ_1_>μ_2_) but not if it is lower. If we use a one-sided test, the critical value in the hypothesis testing is based on the top α percentile from the distribution of the test statistics; if we use a two-sided test, the critical value is the top $\frac{\alpha }{2}$ percentile. Practically, a one-sided test requires a smaller required sample size than a two-sided test (see below).

### Effect size

The effect size is a value that measures the strength of an association that is being claimed. Thus, the effect size is closely related to the statistical test used. For example, if we hypothesize that there is a group difference between the means of a certain biomarker of the disease group and the healthy group, then Cohen's *d* is a commonly used effect size defined as the difference between two means divided by the pooled standard deviation for the data, i.e., $d=\frac{{\bar{x}}_{disease}-{\bar{x}}_{healthy}}{s}$, where *s* is the pooled SD $s=\sqrt{\frac{\left(n_{disease}-1\right)SD_{disease}^{2}+\left(n_{healthy}-1\right)SD_{healthy}^{2}}{n_{disease}+n_{healthy}-2}}$ or in the case of equal sample size $d\;=\;\frac{{\bar{x}}_{disease}-{\bar{x}}_{healthy}}{\sqrt{\frac{SD_{disease}^{2}{+SD}_{healthy}^{2}}{2}}}$. The most critical feature of effect size is that it is not influenced by the sample size. The effect size can usually be calculated using preliminary data observed in a smaller-scale study or in the literature for similar studies. In practice, if practitioners have experience with the biomarker, then it is helpful to define a clinically relevant effect size based on experience. If there is no historical data or experience with the biomarker at hand, Cohen and Sawilowsky (1, 2) laid out a general rule of thumb on the magnitudes of *d* = 0.01 to 2.0, with small (*d* = 0.2), medium (*d* = 0.5), large (*d* = 0.8), and huge (*d* = 2) effect sizes (see Supplementary material 1). When we compare the proportions in two groups, which can also be considered as comparing means of binary outcomes in two groups, the effect size and hence sample size can be calculated by similar metrics designed specifically for proportions, such as Cohen's h or Cohen's ω (1).

If there is another type of association or hypothesis to be used, e.g., for comparing the means of multiple groups, a different type of effect size should be chosen, which we will briefly discuss in a later section.

### Relating the statistical testing and sample size calculation

In a simplified setting of *n*_*disease*_ = *n*_*healthy*_ = *n*, we could roughly write the required sample size as $n\approx {\left(\frac{Z_{1-\frac{\alpha }{2}}+Z_{1-\beta }}{d}\right)}^{2}*2$ for a two-sample two-sided *t*-test at the significance level of α with power 1−β, where $Z_{1-\frac{\alpha }{2}}$ and *Z*_1−β_ are the ($1-\frac{\alpha }{2}$)-th and (1−β)-th percentile of a standard normal distribution (for more detailed calculations, see Supplementary material 2). Here, we use approximation, so this formula may slightly underestimate the required sample size. Then, we round up the *n* to the next smallest integer. Using this simplified formula, we note a few generally true and useful relationships: (1) The required sample size is negatively related to the effect size, i.e., in order to detect a smaller effect size, we need a larger sample size; (2) if we decrease the pre-set tolerated type I (α) and type II error (β), or increase the intended power (1−β), then the required sample size is also larger; (3) in practice, we usually set up α, β, and effect size *d* and calculate the required sample size *n*; however, it is also possible to set up α, β, and the available sample size *n*, calculate the detectable effect size *d*, and compare this detectable effect size to the clinically or practically relevant effect size.

### Expected attrition and corrected sample size

The calculated required sample size is the minimum number needed to achieve the pre-set parameters. In practice, there is oftentimes dropout throughout the study period. For example, if we expect a 10% dropout or attrition rate, then our final corrected sample size will be the minimum required sample size divided by 0.9 = 90% (=100%−10%).

### Allocation ratio

Although random assignment to experimental groups in animals or treatment arms in humans on a 1:1 basis has long been the standard (3), alternative allocation ratios such as 2:1 or 3:1 might be employed, where two or three individuals receive a drug for each individual enrolled receiving a placebo. This is usually done in humans to improve overall enrollment given patient demand to increase their likelihood to receive a study drug, or these alternative allocation ratios might be employed to learn more about the pharmacokinetics and adverse effects of a drug (4). However, a 2:1 allocation ratio requires 12% more subjects, and a 3:1 allocation ratio requires 33% more subjects than a 1:1 allocation ratio to detect the same size effect with equivalent power (3) (also see Supplementary material 2 for justification).

### Other types of tests and power calculation

For the discussions above, we mainly focused on comparing the means of the two groups. If we have other scientific questions, e.g., comparing the means of a continuous variable in more than two groups, investigating the association between two continuous variables, and exploring the explained variance in multiple regression, then the corresponding tests we use are the F test for analysis of variance (ANOVA), the Z test for Pearson correlation coefficient, and the F test based on the R^2^ of a multiple regression model. The corresponding effect sizes for the F test and Pearson correlation coefficient are Cohen's f^2^ and the Pearson correlation coefficient R, respectively (5). We can develop similar formulas for calculating the required sample size to detect the given effect sizes.

### Software

There is a multitude of appropriate programs to calculate sample sizes, including G^*^Power (6), R statistical software (7), Epitools (8), OpenEpi (9), and Biomath (10). A simple and intuitive program is G^*^Power (6), which we will use below to illustrate our animal and human examples of sample size calculation. As an alternative, we will provide the R codes (7) for the same calculations in Supplementary material 3.

## Animal studies

**Figure 1:**
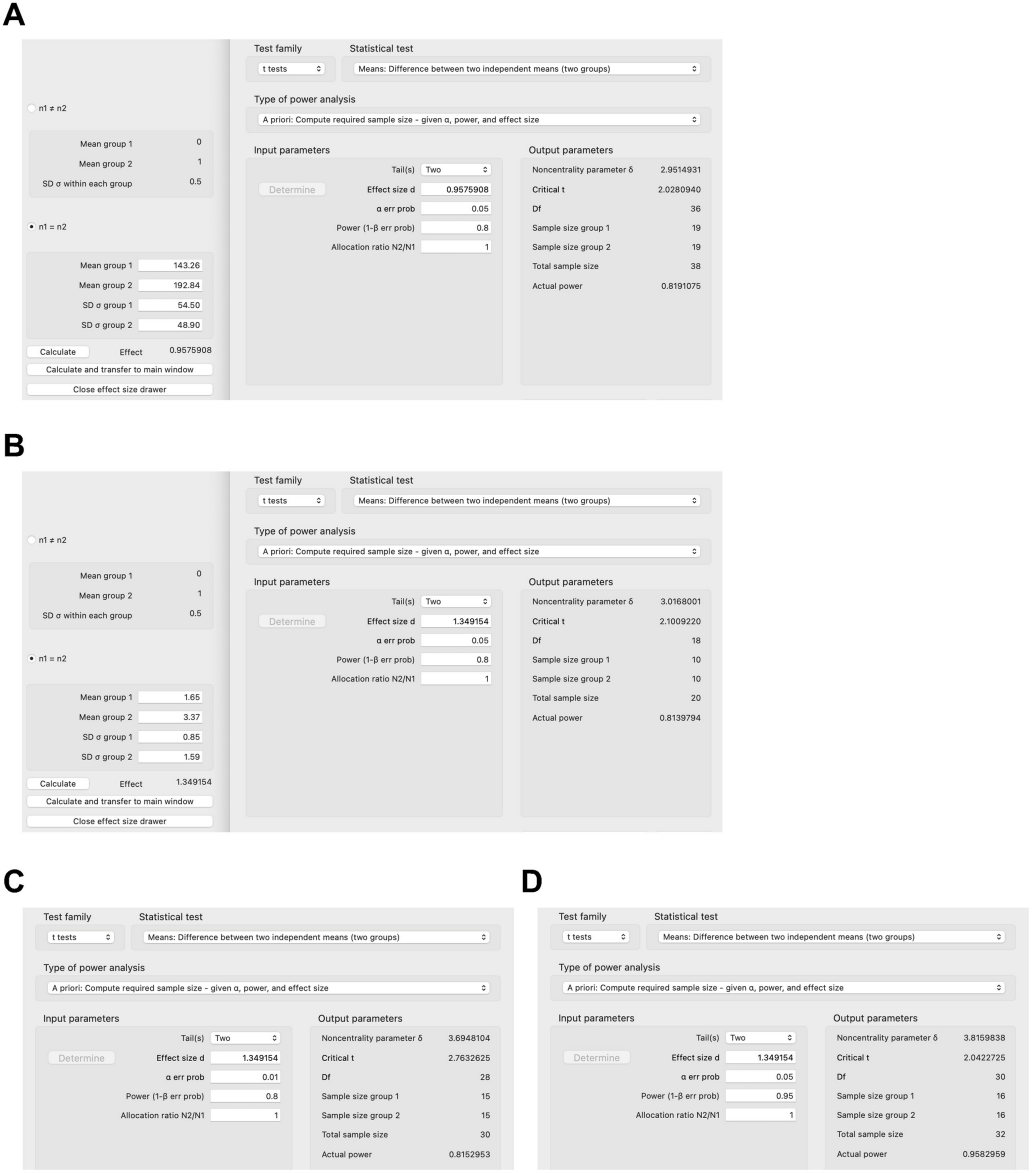
Sample size calculations for select animal studies using G*Power. **(A)** Sample size calculation based on hepatic triglyceride concentration in colesevelam-treated and Western diet-fed mice with type I error of 0.05 and power of 0.8. **(B–D)** Sample size calculation based on gene expression of inflammatory marker tumor necrosis factor (TNF) in the liver in colesevelam-treated and Western diet-fed mice with **(B)** type I error of 0.05 and power of 0.8, **(C)** type I error of 0.01 and power of 0.8, or **(D)** type I error of 0.05 and power of 0.95.

In this section, we will provide practical examples of sample size calculation for animal studies. In order to estimate the sample size for an animal study, one of the more difficult components is to determine the effect size. The effect size depends on the respective outcome the researcher wants to examine. For example, in a mouse model of Western diet-induced liver disease, one of the more important outcomes is the liver triglyceride concentration (11). If a researcher aims to investigate the effect of a drug, e.g., a bile acid binder, on diet-induced liver disease, he/she can attempt to extrapolate outcomes—and hence the expected effect size—from a study similar to his/her proposed project. The bile acid binder colesevelam decreases the hepatic triglyceride concentration to 143.26 mg/g liver weight (standard deviation [SD] 54.50 mg/g) in mice after Western diet feeding compared with 192.84 mg/g (SD 48.90 mg/g) in the Western diet-fed group not treated with the bile acid sequestrant (11). The effect size can be calculated with G^*^Power (6), other software, or manually (1): Cohen's $d\;=\;\frac{{\bar{x}}_{western\;diet}\;-\;{\bar{x}}_{western\;diet\;plus\;colesevelam}}{\sqrt{\frac{SD_{western\;diet}^{2}{\;+\;SD}_{western\;diet\;plus\;colesevelam}^{2}}{2}}}\;=\;\frac{192.84\;-\;143.26}{\sqrt{\frac{48.9^{2}+54.5^{2}}{2}}}\;=0.96$ (Figure 1A). With a two-tailed calculation and an effect size of 0.96, type I error of 0.05, power of 0.8, and an allocation ratio of 1:1, the raw sample size per group of the proposed new bile acid binder experiment is 19, and—with an attrition of 10%—the corrected sample size per group is 22 (19/0.9=21.11) (Figure 1A). However, if another outcome is being chosen, such as markers for liver inflammation, e.g., gene expression of tumor necrosis factor (TNF), with 1.65 relative units (SD 0.85) in the colesevelam-treated group vs. the untreated group with 3.37 (SD 1.59), the effect size is much higher at 1.35, resulting in a lower sample size of 10 per group (Figure 1B) and a corrected sample size of 12 per group to account for 10% expected attrition (10/0.9 = 11.11). This shows that the calculated sample size depends markedly on the selected outcome variable. Furthermore, decreasing the tolerated type I error (e.g., from 0.05 to 0.01) or increasing the power (e.g., from 0.8 to 0.95) increases the required sample size per group (e.g., from 10 to 15 or from 10 to 16, respectively, Figures 1C, D).

In addition to extrapolating expected results to similar experimental environments, calculating the sample for a larger experiment based on a pilot experiment with a small sample size can also be done. If a pilot experiment over 9 months showed that a certain drug decreased the tumor growth in five rats (4, 3, 6, 4, and 4 tumors/rat respectively; mean 4.2 tumors/rat, SD 1.10) versus five control rats (6, 5, 4, 7, 5 tumors/rat, respectively; mean 5.4, SD 1.14, *p* = 0.13 Student's *t*-test), the effect size is 1.07, and the calculated total sample size for a larger experiment is 15 rats per group using a two-tailed analysis. In this case, a one-tailed analysis could also be used, since the pilot experiment suggests that the drug might be protective against tumor growth and the follow-up experiment would focus rather on whether the drug truly significantly reduces the tumor burden relative to controls and not as well whether controls will have a significantly lower tumor burden than the drug group. The calculated sample size per group would be 12 rats per one-tailed analysis, potentially markedly decreasing the costs for maintenance of the rodents over long experimental periods such as 9 months compared with 15 rats per group per two-tailed analysis (prior to correcting for attrition).

Animal models oftentimes include four groups (12–14), two of which might be on a special diet (or have a specific genotype), and the other two groups are on a control diet (or are wild-type mice, etc.). Furthermore, one group of the special diet groups and one group of the control diet groups might then be treated with a drug, and the other two groups are not. It is of major interest to know if the drug improves a certain disease induced by the special diet compared with the other group fed the special diet but not treated with the drug. However, the question might sometimes be posed by reviewers of submitted manuscripts or grants what the most appropriate sample size of the control animals is, that is, the two groups on the control diet, which are not of primary interest. Many articles commonly use five rodents only or even fewer for those control groups, in particular in those rodent models that cause a stark disease phenotype due to a special diet or genotype or similar conditions (13, 15–18). A mouse model of high-fat diet-induced obesity might serve as an example, in which mice gained 15.75 g on average over 16 weeks on a high-fat diet (SD 7.63) vs. 2.5 g (SD 2.65) in control mice on a control diet (17). The estimated uncorrected sample size in the control group would be 3 and 9 in the high-fat diet group using a 3:1 allocation ratio and a two-tailed analysis. A one-tailed analysis would provide an uncorrected sample size of 2 in the control group and 6 in the high-fat diet group using a 3:1 allocation ratio. Clear phenotypes can be achieved with rodent models of diet-induced metabolic diseases including type 2 diabetes (19) or non-alcoholic steatohepatitis (20) as well as chemically induced diseases [e.g., dextran sulfate sodium-induced colitis (21)] or (at least partially) genetic diseases [such as Alzheimer's disease (18) or autism (22)]. In instances like these with established rodent models and distinct phenotypes, untreated control groups of five or fewer rodents are acceptable with or without power calculations as it is rather of primary interest if an intervention (such as a drug) changes the phenotype in the experimental group compared with an experimental group without the drug. On the other hand, it is of utmost importance that a power calculation be carried out for these experimental groups, as described above.

## Human studies

The sample size can be calculated for human studies analogous to mouse studies. For example, drug A may decrease the inflammatory marker fecal calprotectin in humans with inflammatory bowel disease by 170 mcg/g (standard deviation 150 mcg/g) vs. 90 mcg/g (SD 100 mcg/g) in the placebo group in a small pilot. The effect size will be 0.63 with 0.05 type I error, 95% power, and 1:1 allocation ratio, and this will require 67 subjects per group (or 75 subjects after accounting for 10% attrition) for the larger randomized controlled trial. However, it can be difficult to calculate the effect size in human studies if no pilot studies have been done. In those cases, one can usually estimate effect sizes as small (*d* = 0.2), medium (*d* = 0.5), or large (*d* = 0.8), as suggested by Cohen and Sawilowsky (1, 2). This also highlights that the effect sizes in human studies are usually much smaller, and the sample sizes are usually much larger than in animal studies (the aim for effect sizes in animal studies is generally >1.0, see above).

**Figure 2:**
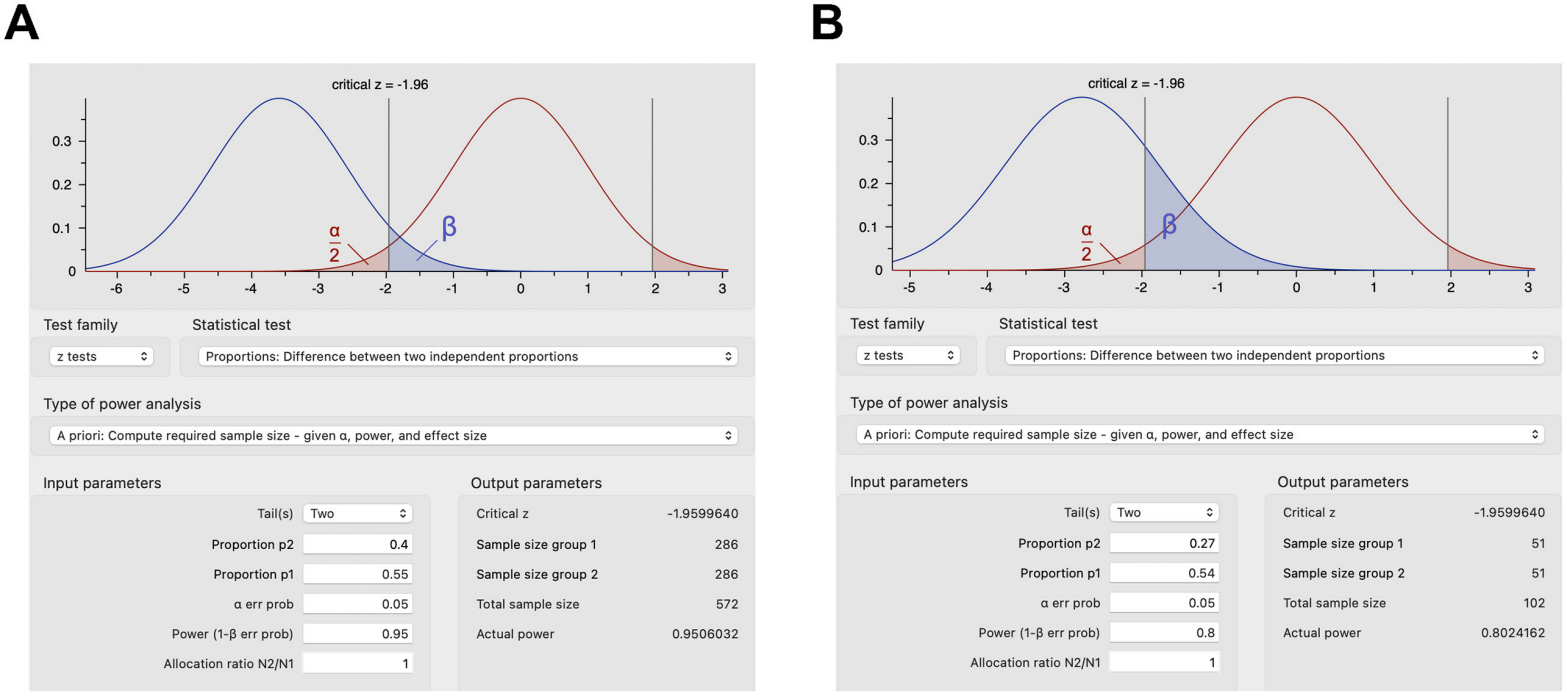
Sample size calculations for select human studies using G*Power. **(A)** Sample size calculations based on expected proportions of response for rifaximin vs. placebo in irritable bowel syndrome with type I error of 0.05 and power of 0.95. **(B)** Sample size calculations based on the expected incidence of postoperative acute kidney injury with dexmedetomidine infusion vs. placebo with type I error of 0.05 and power of 0.8.

However, proportions are more commonly used to calculate sample sizes in human studies (23–25). In a human trial of rifaximin in irritable bowel syndrome, the sample size was calculated using the difference between two independent proportions (23). An improvement was estimated *a priori* in 55% of the rifaximin group and in 40% of the placebo group, which with 95% power and a significance level of 0.05 would require ~300 subjects per group (23), or more accurately 286 subjects per group per z test (Figure 2A), plus 16 or 32 subjects per group corrected for 5% or 10% attrition (286/0.95 = 301.05 or 286/0.9 = 317.78), respectively. Effect sizes and proposed sample sizes can be arbitrary in human studies (26). However, as described above, it is recommended to base estimates on smaller pilot studies investigating the same drug or larger randomized controlled trials scrutinizing a similar drug in the same clinical context or the same drug in a slightly different clinical context. For example, a human study examined the effect of dexmedetomidine on acute kidney injury after aortic surgery (25), basing the estimated 54% incidence of postoperative acute kidney injury on a prior study (27) and estimating that the dexmedetomidine infusion would decrease the incidence of postoperative acute kidney injury by half to 27% similar to a study on acute kidney injury following valvular heart surgery (28). These proportions with a statistical power of 80% and type I error of 0.05 provide a sample size of 51 subjects per group (Figure 2B) or 54 subjects per group after correcting for 5% attrition (25).

## Conclusion

In conclusion, the appropriate calculation of the required sample size is central when designing animal or human studies for a variety of reasons, such as ethical considerations, decreasing costs, time, effort, and the use of other resources.

## Author contributions

XZ and PH conceived and designed the study, performed the statistical analysis, and wrote the first draft of the manuscript and edited the manuscript. All authors approved the submitted version.
