# Supplementary material for: How to calculate sample size in animal and human studies
Source: Front Med (Lausanne). 2023 Aug 17;10:1215927. doi: 10.3389/fmed.2023.1215927 (PMC10469945; doi:10.3389/fmed.2023.1215927)
Supplement: Supplementary file 1 [file Data_Sheet_1.ZIP › Supplementary Material.docx]

Supplementary Material

Supplementary Material 1

Supplementary Material 1: Illustration of small ($d$=0.2), medium ($d$=0.5), large ($d$=0.8), and huge ($d$=2) Cohen’s d values using Gaussian densities.

Supplementary Material 2

**Relating the statistical testing and sample size calculation**: Suppose in the example of two sample comparisons, we set up the null hypothesis ($H_{0}$) and alternative hypothesis ($H_{1}$) as:

$H_{0}:\delta=0$ vs $H_{1}:\delta=\delta_{0}\neq0,$

where the $\delta$ is the mean difference between the disease and healthy control groups. And we estimate this difference via the sample mean difference $\hat{\delta}=\bar{x}_{disease}-\bar{x}_{healthy}$. The test statistic is $t=\frac{\hat{\delta}-0}{s\sqrt{\frac{1}{n_{disease}}+\frac{1}{n_{healthy}}}}$, where $s$ is the pooled standard deviation introduced in the main manuscript. Suppose for simplicity, we let $n_{disease}=n_{healthy}=n$. If |$t|>t_{1-\alpha/2,2n-2}$, then we reject the $H_{0}$ and conclude that there is a significant difference between the two groups. The focus of power calculation is when the alternative is true (say $\delta_{0}>0$) and the $\hat{\delta}$ follows a t distribution of center $\delta_{0}$. Suppose we have $\delta_{0}>0$. Then, by the definition of $\alpha$ and $\beta$, we have

$P\left( \frac{\hat{\delta}}{s\sqrt{\frac{2}{n}}}>t_{1-\frac{\alpha}{2}, 2n-2} \right)+P\left( \frac{\hat{\delta}}{s\sqrt{\frac{2}{n}}}<{-t}_{1-\frac{\alpha}{2}, 2n-2} \right)=1-\beta$ , and

$P\left( \frac{\hat{\delta}-\delta_{0}}{s\sqrt{\frac{2}{n}}}>t_{1-\frac{\alpha}{2}, 2n-2}-\frac{\delta_{0}}{s\sqrt{\frac{2}{n}}} \right)+P\left( \frac{\hat{\delta}-\delta_{0}}{s\sqrt{\frac{2}{n}}}<{-t}_{1-\frac{\alpha}{2}, 2n-2}-\frac{\delta_{0}}{s\sqrt{\frac{2}{n}}} \right)=1-\beta$

Now $\frac{\hat{\delta}-\delta_{0}}{s\sqrt{\frac{2}{n}}}$ follows t distribution with $2n-2$ degree of freedom. We could solve the above equation using numerical method to find the minimum sample size.

Another way to approximate the solution would be to solve [1]:

$\frac{\delta_{0}}{s\sqrt{\frac{2}{n}}} -t_{1-\alpha/2,2n-2}=t_{1-\beta,2n-2}$.

This finally gives:

$d=\frac{\delta_{0}}{s}=\left( t_{1-\alpha/2,2n-2}+t_{1-\beta,2n-2} \right)\sqrt{\frac{2}{n}}$ and $n=2\left( \frac{t_{1-\alpha/2,2n-2}+t_{1-\beta,2n-2}}{d} \right)^{2}$.

If we have unequal sample size, say $\kappa=\frac{n_{disease}}{n_{healthy}}$, then we write $n_{disease}=\kappa n_{healthy}$ to insert in the formula above, we will note that $n_{healthy}=(1+\frac{1}{\kappa})\left( \frac{t_{1-\alpha/2,2n-2}+t_{1-\beta,2n-2}}{d} \right)^{2}$. Using this we could verify that the total required sample size will be ~12% more if $\kappa=2$ and will be ~33% more if $\kappa=3.$

Supplementary Material 3

See the R code file “Supplementary Material 3” for sample size calculations detailed in the manuscript using R statistical software.

References

[1] Chow, S-C, Shao, J., Wang, H., 2008, Sample size calculation in clinical research. CRC press.
